# The earthquake cycle in the dry lower continental crust: insights from two deeply exhumed terranes (Musgrave Ranges, Australia and Lofoten, Norway)

**DOI:** 10.1098/rsta.2019.0416

**Published:** 2021-02-01

**Authors:** Luca Menegon, Lucy Campbell, Neil Mancktelow, Alfredo Camacho, Sebastian Wex, Simone Papa, Giovanni Toffol, Giorgio Pennacchioni

**Affiliations:** 1 The Njord Centre, Department of Geoscience, University of Oslo, 1048 Blindern, Norway; 2 School of Geography, Earth and Environmental Sciences, University of Plymouth, Drake Circus, PL4 8AA Plymouth, UK; 3 Department of Earth Sciences, ETH Zurich, 8092 Zurich, Switzerland; 4 Department of Geological Sciences, University of Manitoba, Winnipeg, Manitoba R3T 2N2, Canada; 5 Department of Geosciences, University of Padova, Via Gradenigo 6, 35131 Padua, Italy

**Keywords:** lower crustal earthquakes, pseudotachylytes, ductile shear zones, transient deformation, dry lower continental crust

## Abstract

This paper discusses the results of field-based geological investigations of exhumed rocks exposed in the Musgrave Ranges (Central Australia) and in Nusfjord (Lofoten, Norway) that preserve evidence for lower continental crustal earthquakes with focal depths of approximately 25–40 km. These studies have established that deformation of the dry lower continental crust is characterized by a cyclic interplay between viscous creep (mylonitization) and brittle, seismic slip associated with the formation of pseudotachylytes (a solidified melt produced during seismic slip along a fault in silicate rocks). Seismic slip triggers rheological weakening and a transition to viscous creep, which may be already active during the immediate post-seismic deformation along faults initially characterized by frictional melting and wall-rock damage. The cyclical interplay between seismic slip and viscous creep implies transient oscillations in stress and strain rate, which are preserved in the shear zone microstructure. In both localities, the spatial distribution of pseudotachylytes is consistent with a local (deep) source for the transient high stresses required to generate earthquakes in the lower crust. This deep source is the result of localized stress amplification in dry and strong materials generated at the contacts with ductile shear zones, producing multiple generations of pseudotachylyte over geological time. This implies that both the short- and the long-term rheological evolution of the dry lower crust typical of continental interiors is controlled by earthquake cycle deformation.

This article is part of a discussion meeting issue ‘Understanding earthquakes using the geological record’.

## 1. Introduction

Some 20% of intracontinental earthquakes of moment magnitude (*M*_w_) > 5 nucleate in the middle to lower crust at focal depths of 20–40 km [1,2]. For example, in the Himalaya a significant proportion of seismicity, including aftershocks associated with the destructive 2001 Bhuj earthquake in India, nucleated in the lower crust of the Indian shield underthrusting Tibet [1,3]. Likewise, crustal earthquakes at focal depths of 20–30 km regularly occur beneath the northern foreland of the Central Alps [4]. Lower crustal earthquakes are also frequent in active rifts (e.g. Bajkal rift: [5]; East African rift: [6]) and along major strike slip faults (e.g. North Anatolian Fault: [7]).

The physical mechanisms that initiate lower crustal earthquakes are not well understood, as the lower crust is expected to be rheologically weak and to deform by distributed viscous flow at the high ambient *P*–*T* conditions ([2] and refs. therein). However, the anhydrous conditions and the lack of grain boundary fluids in the lower crust inhibit crystal plastic deformation and diffusive mass transfer, resulting in a lower crust with high viscosity and high mechanical strength [8–14]. Thus, there is general consensus that a strong, seismogenic lower crust reflects the rheology of anhydrous mineral assemblages, which are typically found in granulite facies rocks [15,16].

The generation of earthquakes in the lower crust remains an intensely debated issue, as it requires mechanisms capable of developing, at least transiently, very high differential stresses. Transient seismic fracturing at these crustal levels has been attributed to the downward propagation of seismic ruptures from the overlying brittle, seismogenic upper crust [17–19]. Stress transfer from seismic faulting in the upper crust can induce a transient abrupt deepening of the frictional–viscous transition below the lower termination of the fault [20], and many lower crustal aftershocks have been interpreted in this way [21,22]. However, the downward rupture propagation and the stress transfer models are difficult to reconcile with deep intracontinental earthquakes that occur far from fault systems where shallow crustal seismicity is focused. For example, lower crustal seismicity up to *M*_w_ 4 in the Alpine northern foreland occurs in a region that does not host shallow seismicity [4]. Thus, alternative mechanisms by which earthquakes can nucleate in the lower crust must be invoked.

In the absence of mechanical perturbations originating at shallower crustal levels, local weakening mechanisms that could facilitate brittle failure and explain earthquake generation in the lower crust include dehydration reactions, leading to increased fluid pressure and/or local stress redistributions, and eclogitization reactions [23–26]. ‘Metamorphic earthquakes' and increased fluid pressure require, however, syn-deformational reactions or local fluid-rich conditions that may not occur in all locations hosting lower crustal seismicity.

An alternative proposal to brittle rupture has been thermal runaway, where thermal feedback in highly localized ductile shear zones leads to rapid slip and melting. Plastic deformation instabilities and shear-induced melting have long been considered a plausible explanation for intermediate-depth and deep earthquakes and for the cyclic generation of pseudotachylytes in shear zones [27–30]. This process was further investigated by numerical models for both mantle and crustal rheologies [31–41], seismological studies [42,43] and experiments [44], showing that pseudotachylytes can develop due to the positive feedback between shear heating and strain rate, eventually leading to a catastrophic seismic slip failure and melting (thermal runaway). This may occur at realistic background flow stresses in a shear zone [38,41], provided that grain size reduction occurs prior to thermal runaway. However, although some cases of middle and lower crustal pseudotachylyte/mylonite associations have been interpreted as being due to plastic instabilities [30,37,45–48], unambiguous microstructural evidence for this process is still missing [41].

Direct investigations of exhumed lower crustal sections have provided new insights into the seismic activity and the rheology of these deeper crustal levels. These field investigations have consistently demonstrated that the deformation of the dry lower continental crust is characterized by a cyclic interplay between viscous creep (mylonitization) and brittle, seismic fracturing associated with the formation of pseudotachylytes [14,49–55]. A picture is emerging in which a seismically active lower crust facilitates metamorphic and rheological transition in otherwise dry, strong and metastable rocks [12,14,56]. Dynamic rupture propagation and seismic slip may trigger grain size reduction, fluid infiltration, weakening and a transition to aseismic, viscous creep along faults initially characterized by fracturing, frictional melting and wall-rock damage [14,57–59].

**Figure 1.**
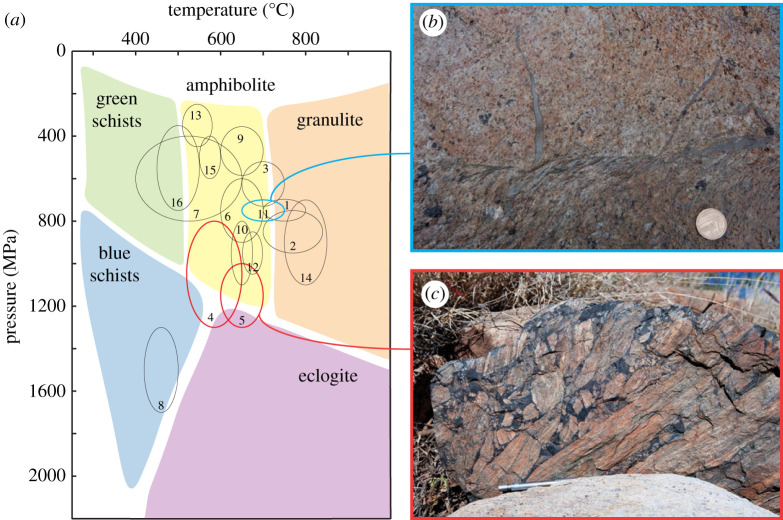
(*a*) Pressure–temperature (*P*–*T*) diagram showing occurrences of associations of coeval pseudotachylytes and mylonites listed in table 1. Standard uncertainties of 0.2 GPa and 50°C have been added if ranges are not reported in the original reference. Numbers from 1 to 16 refer to the list order in table 1, facies diagram redrawn from Winter [77]. (*b*,*c*) Field examples of pseudotachylytes from the selected localities discussed in this paper: (*b*) mylonitized pseudotachylyte with relict undeformed injection veins crosscutting non-foliated gabbronorite from Nusfjord, Lofoten [52] and (*c*) pseudotachylyte breccia from the Musgrave Ranges [63]. (Online version in colour.)

A few regions worldwide expose outstanding natural laboratories that enable direct observations of seismic–aseismic deformation cycles in the lower crust. These localities are reported in table 1 and in figure 1, together with estimates of the *P*–*T* conditions of pseudotachylyte generation and the interpretation for their origin [19,41,45,46,50,52–54,60–76].

**Table 1.** Occurrences of associations of coeval pseudotachylytes and mylonites significantly below the brittle–ductile transition.

location	*T* range (°C)	*P* range (MPa)	depth (km)	context	host rock lithology	proposed mechanisms
1. Cora Lake shear zone, Canada [19]	700–800	700–800	24–28	continental crust	metagabbro	plastic instability/interaction with an overlying brittle fault system
2. Minas Fault Zone, Canada [46]	700–860	750–950	28–35	continental crust	mafic granulite	plastic instability
3. MacRobertson Land, Antarctica [60]	700	520–730		continental crust	charnockite	brittle fracturing caused by work hardening in shear zones
4. Woodroffe thrust, Australia [54,61,62]	520–650	800–1300	32–40	continental crust	quartzofeldspathic gneiss, some mafic gneiss and granite	downward migration of earthquake form the upper seismogenic layer which can cause a strain rate increase in the lower crust locally triggering new earthquakes
5. Davenport shear zone, Australia [53,63]	600–700	1000–1300	33–43	continental crust	quartzofeldspathic gneiss, mafic intrusions	stress localization due to jostling of less deformed strong blocks within the irregular shear zone network
6. Azul Megashear Zone, Argentina [64]	600–700	600–900		continental crust	migmatitic gneiss and granite	intermittent seismic/aseismic slip
7. Dahezhen shear zone, China [65]	400–650	400–800	20–33	continental crust	granitic and quartz-monzonite gneiss	downward propagation of dynamic fracture
8. Cima di Gratera, Corsica [66–69]	470	1500	60–90	oceanic crust and mantle	metagabbro, peridotite	thermal runaway [69]/asperities in intra-slab fault [68]
9. Eidsfjord, Lofoten, Norway [70]	650	455–685	17–25	continental crust	mangerite, monzonites, anorthosites and granitic gneiss	dynamic downward rupture
10. Fiskefjord, Lofoten, Norway [70,71]	625–675	800–1100	32–50	continental crust	mangerite, monzonites, anorthosites and granitic gneiss	dynamic downward rupture
11. Flakstadøy, Lofoten, Norway [52,55,72]	650–750	700–800	>25, >45 (in eclogite shear zones)	continental crust	anorthosites, gabbros, mangerites and charnockites	stress concentration in less deformed strong blocks due to active viscous deformation across shear zone network [55]
12. Serre massif, Italy [73]	650–700	>850	>31	continental crust	felsic and mafic granulites	ductile flow with intermittent high strain rate episodes
13. Mont Mary nappe, Italy [41,50]	510–580	250–450	10–20	continental crust	metapelites	transient brittle instabilities due to strain hardening in water-deficient conditions [50]/downward propagation of stress [41]
14. Balmuccia massif, Ivrea zone, Italy [74]	800	700–1100		subcontinental mantle	utramafic	strain hardening due to reduction of H_2_O activity
15. Premosello, Ivrea zone, Italy [75]	550–600	400–600	15–20	continental crust	metagabbro and felsic mylonites	downward propagation of seismic fractures
16. Outer Hebrides Fault Zone, Scotland [45,76]	500	550	15–25	continental crust	quartzofeldspathic gneiss	stress concentration at strong lensoid-shaped bodies [76]/plastic instabilities [45]

This article reviews recent developments in the study of the interplay between brittle seismic fracturing and viscous deformation in the relatively dry lower crust, and argues that the earthquake cycle controls the short- and long-term rheological evolution of the granulitic lower crust typical of continental interiors. We focus on exhumed networks of coeval pseudotachylytes and mylonites from the Musgrave Ranges (central Australia) and from Nusfjord (Lofoten, Norway). We first present and discuss observations from the Musgrave Ranges as an example of an almost unique record of a well-exposed crustal-scale section of the seismic structure of the middle to lower continental crust. We then present and discuss detailed meso- and microscale observations from Nusfjord that provide an exceptional opportunity to investigate transient deformation during the earthquake cycle in the lower crust.

## 2. The Musgrave Ranges, central Australia

The EW-trending Musgrave Province is a Mesoproterozoic, granulite to amphibolite facies terrane covering an area of about 120 000 km^2^ in the centre of the Australian continent. The Province was heterogeneously overprinted by high-strain deformation during the Petermann Orogeny (*ca* 630–520 Ma [78,79]). The oldest rocks are predominantly felsic orthogneisses, with *ca* 1600–1540 Ma protolith ages (e.g. [78,80–83]), unconformably overlain by Late Mesoproterozoic (*ca* 1.4 Ga maximum age) metasedimentary rocks [84,85]. The most widespread tectono-thermal event in the Musgrave Province is the *ca* 1220–1150 Ma Musgravian Orogeny, which produced high- to ultrahigh-temperature (approx. 900°C) granulites [86,87] and voluminous anhydrous syn- to post-tectonic granites (Pitjanjatjara Supersuite; [88]). Pressure estimates of the metamorphism, in the range of 0.6–0.8 GPa [82,86,87], indicate mid-crustal conditions across the entire Province during the Musgravian Orogeny. The Musgravian metamorphic imprint of the rocks now exposed in the Musgrave Ranges played a fundamental role in determining their rheology during the subsequent Petermann Orogeny. In the period between the Musgravian and the Petermann Orogenies, two major dolerite dyke swarms were emplaced in the Province: (i) the Alcurra swarm at *ca* 1080 Ma [89–91] and (ii) the Amata swarm at *ca* 800 Ma [90,92]. These dykes were not deformed or metamorphosed prior to the subsequent Petermann Orogeny.

### (a) High-strain deformation and metamorphism during the Petermann Orogeny

**Figure 2.**
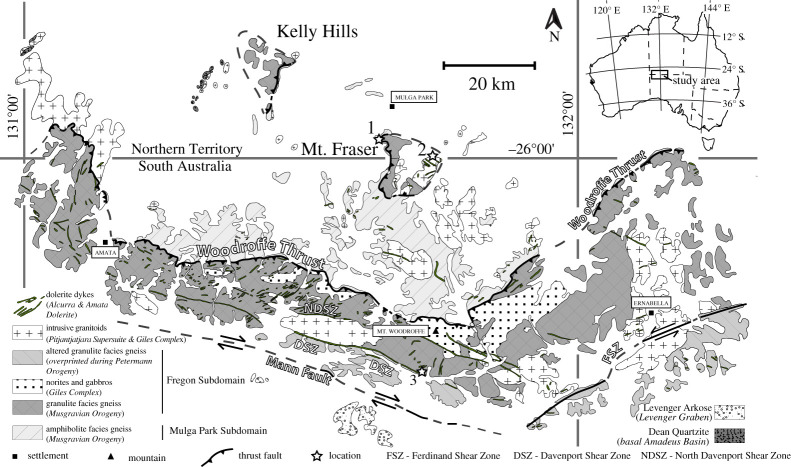
Geological map of the study area in the Musgrave Ranges, modified after Wex *et al*. [62,93], showing the major shear zones and locations of the figures. For more detailed descriptions of the units marked on the map, see Wex *et al*. [62].

A series of major shear zones developed during the Petermann Orogeny (figure 2): the Woodroffe Thrust, the northern and southern Davenport Shear Zone (DSZ), the Mann ‘Fault’ (also a shear zone) and the Ferdinand Shear Zone (FSZ) [61,62,78,83,94–101]. The presently exposed portions of these regional structures were active at middle to lower crustal conditions and host the largest known occurrences worldwide of pseudotachylyte broadly coeval with high-grade mylonite [53,61–63,93,102]. The Woodroffe Thrust [94–96] is a crustal-scale structure that extends east-west over more than 600 km, with a generally shallow to moderate southerly dip (approx. 30°) and a top-N relative movement of more than 60 km [62]. It produced a telescoped, *ca* 40 km thick, section through the continental crust that is now exposed. The Woodroffe Thrust juxtaposes units with distinctly different Musgravian metamorphism [78,94,95,97,98,103]: (i) the hanging wall Fregon Subdomain that was thoroughly dehydrated under granulite facies conditions; and (ii) the footwall Mulga Park Subdomain that only reached amphibolite facies conditions and contains more hydrous minerals. This difference in the degree of devolatilization is clearly reflected in the regional maps of thorium concentration determined by airborne gamma-ray surveys, with a distinct jump in concentration across the Woodroffe Thrust from lower values in the hanging wall to higher values in the footwall [63,93]. Except for the difference in Musgravian peak metamorphism, the structural and magmatic histories in the two subdomains prior to their juxtaposition are similar, which suggests that the two units represent different crustal levels of the same terrane [78,100]. Wex *et al*. [93] used the thorium concentrations across the Woodroffe Thrust to establish that the hanging-wall-derived mylonites generally represent less than 10% of the entire width of the shear zone. In general, the thickness of the Woodroffe Thrust mylonites decreases with decreasing metamorphic grade, and increasing availability of aqueous fluids, in the northward direction of thrusting. This ‘inverted’ strain distribution for a thrust, with a large part of the ductile deformation affecting the footwall rocks, is explained by the weaker rheology of the wetter footwall compared to the drier hanging wall [61,96]. The decrease in thickness of the footwall mylonites toward the north can be explained by localization favoured by weakening associated with the relatively more hydrous conditions [104], although quartz piezometry indicates higher synmylonitic differential stress in the north, consistent with the decrease in temperature in the direction of thrusting [93]. Conversely, pseudotachylytes occur mainly in the hanging wall and are generally subordinate or absent in the footwall, especially in the northernmost wetter, cooler and shallower portions.

The Petermann Orogeny resulted in a heterogeneous distribution of shear zones in both subdomains. Large areas of low strain still preserve the Musgravian fabric and metamorphic assemblage in the gneisses and magmatic textures in the syn- to post-Musgravian intrusions. This is especially true of large parts of the Fregon Subdomain north of the Mann Fault (figure 2). A broad zone of distributed shearing is represented by the DSZ (figure 2), with more localized shearing on the North Davenport Shear Zone (NDSZ, figure 2) and FSZ (figure 2). However, in the Fregon Subdomain north of the NDSZ, the pre-Petermann fabric is largely preserved. Dolerite dykes of the Alcurra and Amata swarms are discordantly transected by the pseudotachylyte-rich zone in the immediate hanging wall of the Woodroffe Thrust. Deformation is heterogeneously but more widely distributed in the footwall Mulga Park Subdomain, with the regional development of open folds.

Recrystallization and metamorphic re-equilibration is mainly associated with deformation, whereas in low-strain domains the metamorphic transformation is non-existent or incipient. The dominant mylonitic granitoids, felsic granulites and felsic pseudotachylytes have a synkinematic metamorphic assemblage (decreasing modal abundance from left to right) of quartz + plagioclase + K-feldspar + calcium-rich garnet + biotite ± ilmenite ± hornblende ± magnetite ± calcite. Kyanite, clinopyroxene and rutile are present as additional phases in both the hanging wall and footwall of the southern Woodroffe Thrust, whereas epidote, muscovite, titanite and (rare) chlorite appear in the northern footwall. Low-strain felsic domains show static incipient overprint compatible with the assemblage in the mylonites including (i) neo-crystallized calcium-rich garnet, (ii) kyanite inclusions in plagioclase and (iii) zoned garnet clasts with higher-calcium rims and lower-calcium cores. Mylonitic dolerite dykes and associated pseudotachylytes show synkinematic mineral assemblage of plagioclase + clinopyroxene + garnet + ilmenite ± rutile ± kyanite ± quartz ± K-feldspar ± hornblende ± biotite, with the amount of newly grown garnet gradually decreasing toward the north. In the northernmost locations, garnet is absent and mylonites in the footwall of the Woodroffe Thrust show a greenschist facies assemblage of plagioclase + biotite + anthophyllite/cummingtonite + chlorite + ilmenite + quartz + titanite ± tschermakitic hornblende ± scapolite ± magnetite. In the northernmost footwall, plagioclase is fractured rather than dynamically recrystallized. Undeformed Alcurra and Amata dolerites also locally show incipient metamorphic reactions in the southern areas, with the development of either hornblende or orthopyroxene plus orthopyroxene/spinel symplectite at the contact of magmatic olivine with plagioclase, or pseudomorphic replacement of olivine with orthopyroxene plus orthopyroxene/magnetite symplectite [62]. Neo-crystallized garnet and kyanite inclusions in plagioclase are also common in undeformed, partially recrystallized Alcurra and Amata dolerites in the south.

Available estimates of the metamorphic conditions during the Petermann Orogeny [53,54,62,63,83,93,105] indicate, at the regional scale, a gradual decrease in temperature from 620 to 650°C in the south to approximately 520°C in the north. Pressures of 1.0–1.3 GPa, corresponding to sub-eclogitic facies conditions, typical of the lower continental crust, have been estimated for the south. To the north, the pressure was lower, but less well constrained [62,93]. Generally, dry conditions in the hanging wall (Fregon Subdomain) and southernmost exposed footwall (Mulga Park Subdomain) during the Petermann Orogeny have been inferred from the characteristic presence of kyanite and the absence of epidote within the breakdown products of the anorthite component of plagioclase [63,93]. Kyanite was produced by the metamorphic reaction:

anorthite=grossular garnet+kyanite+quartz, 

and the dominance of this reaction over competing reactions producing zoisite/clinozoisite/epidote implies a water activity less than *ca* 0.004 for the metamorphic conditions in the southern areas [106]. By contrast to the footwall, where a gradient of increasing water is documented toward the north [93], the Fregon Subdomain was dry as a result of the granulite facies Musgravian metamorphism and remained dry during the Petermann Orogeny. The only exception is the area immediately adjacent to the Mann Fault (figure 2), which has been a localized conduit for fluids.

### (b) Distribution of pseudotachylytes

**Figure 3.**
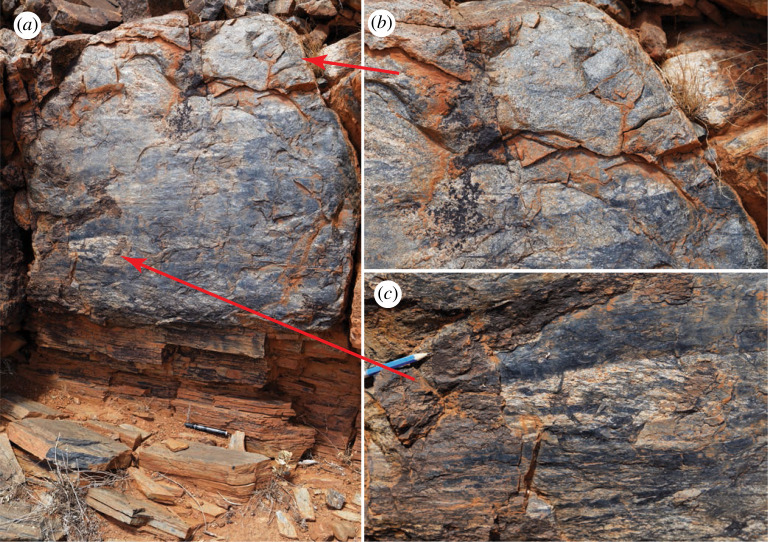
Woodroffe Thrust (*a*), showing little deformed pseudotachylyte breccias with granulite clasts of the Fregon Subdomain (*b*), which become increasingly foliated downward (*c*), with a relatively abrupt transition into mylonites/ultramylonites also derived from the hanging wall. Note the crosscutting, newly developed pseudotachylyte injection vein in (*c*). The contact below to mylonites derived from the footwall Mulga Park Subdomain cannot be directly established in the field but can be determined on the basis of the difference in Th content [93]. View looking E, pen (15 cm in length) aligned parallel to the lineation. Mylonite foliation 188/22 (dip direction/dip); stretching lineation 201/21 (plunge direction/plunge) (corrected to relative to true N). Section below Mt Fraser, Location 1 on figure 2 (GPS location 25° 57′ 34.6′′ S, 131° 38′ 37.3′′ E). (Online version in colour.)

**Figure 4.**
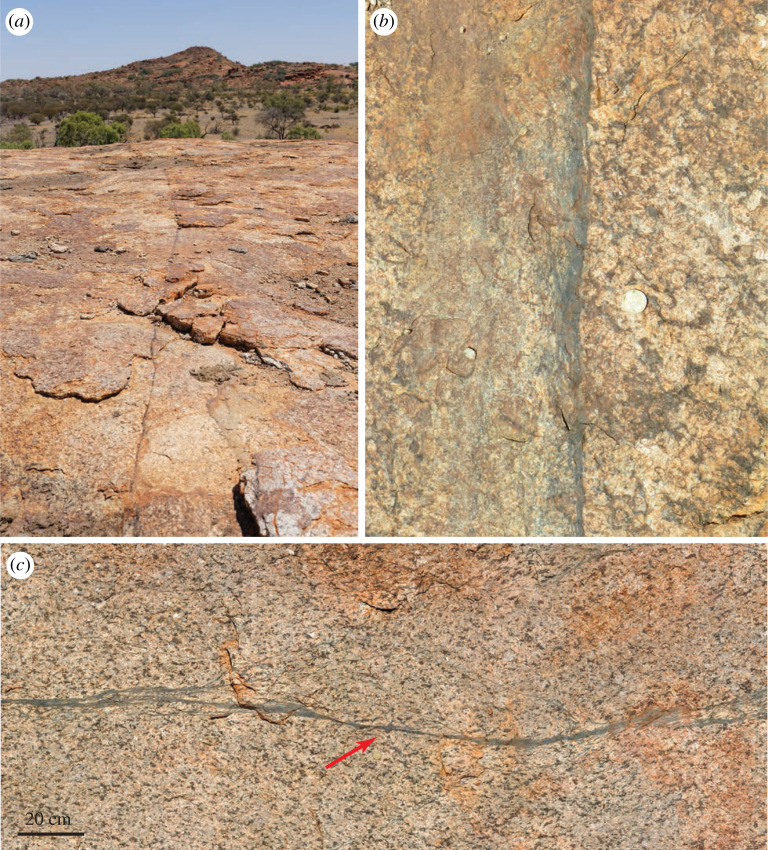
Shear zones in the Fregon Subdomain (hanging wall of the Woodroffe Thrust) localizing on precursor pseudotachylyte, without any evidence for associated hydration. (*a*) Long, narrow shear zone developed in otherwise undeformed meta-granitoid, looking west. (*b*) Close-up of the same shear zone, showing ductile shear localizing on a pre-existing pseudotachylyte. Scale A$2 coin 2.5 cm diameter. GPS location 25° 59′ 50.94′′ S, 131° 44′ 45.07′′ E. (*c*) Shear zone localized on pseudotachylyte without hydration. Note the clearly preserved injection vein near the centre of the mosaic (red arrow). Looking down, top of mosaic toward 320° N. GPS location 25° 59′ 53.16′′ S, 131° 44′ 46.24′′ E. (*a*), (*b*) and (*c*) are all from the immediate area of Location 2 on figure 2. (Online version in colour.)

**Figure 5.**
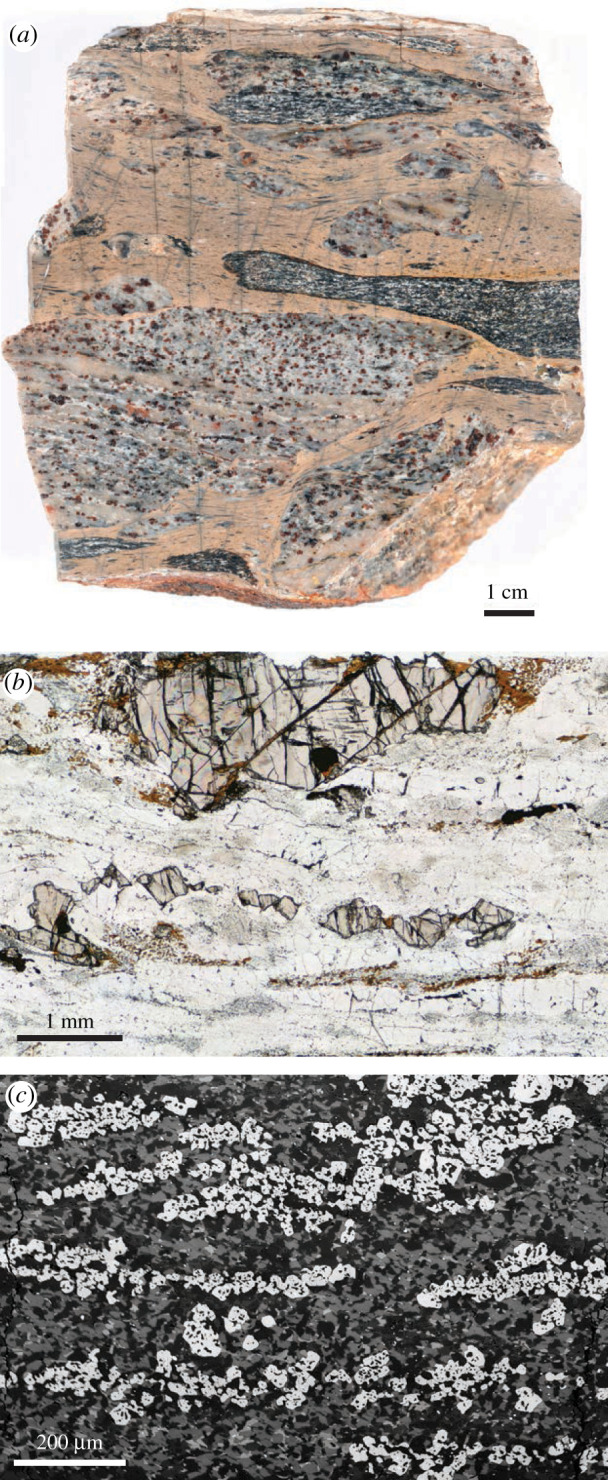
(*a*) Polished slab of a sheared garnet-bearing caramel-coloured pseudotachylyte with clasts of garnet-bearing granulite and a single metabasic clast. Sample F68 of Hawemann *et al*. [63,107]. Note the total lack of hydration of the clasts. (*b*) Photomicrograph from the felsic granulite of (*a*), showing the well-developed foliation and strongly fractured relict granulite facies garnets, which were studied in Hawemann *et al*. [107]. (*c*) Back-scattered environmental scanning electron microscope image of the pseudotachylyte from (*a*), showing the growth of new garnet. This image is a crop from fig. 5b of Hawemann *et al*. [63], which established syn-shearing *P*–*T* conditions for this pseudotachylyte of around 1.05 GPa and 600°C. For imaging conditions, see the original publication. Location 3 on figure 2 (GPS location 26° 23′ 7.09′′ S, 131° 42′ 37.91′′ E). (Online version in colour.)

The Musgrave Ranges are famous for the wide distribution and volume of pseudotachylyte [61,102]. Large amounts of pseudotachylyte characterize the immediate hanging wall of the Woodroffe Thrust over a thickness of a few hundred metres, with the spatial density of pseudotachylyte decreasing away from the thrust contact. The Woodroffe Thrust itself is marked by a major horizon (in some places up to 10 m thick) of hanging wall pseudotachylyte breccias that are strongly mylonitized in a few metres thick zone at the immediate contact with the footwall mylonites (figure 3*a*). These sheared pseudotachylytes are in turn overprinted by undeformed pseudotachylyte injection veins (figure 3*c*). Other several-metre-thick horizons of pseudotachylyte breccias and of spatially dense networks of pseudotachylyte occur subparallel to the thrust surface further within the hanging wall, but with generally decreasing spatial frequency and abundance [61,62,102]. Above the upper boundary to the Woodroffe Thrust, the fabric of the Musgravian granulites is undisturbed and Alcurra and Amata dolerite dykes are transected at a high angle. Within these undeformed granulites, there are scattered occurrences of pseudotachylyte with no obvious trend in the amount developed from south to north. In the high-pressure DSZ (figure 2), pseudotachylyte developed repetitively under the ambient dry lower crustal conditions (1.0–1.3 GPa, 620–650°C; [63]). There is no evidence of the introduction of water during seismic fracture and pseudotachylyte formation or during subsequent localization of ductile shear on the pseudotachylyte (figures 4 and 5). In the footwall, pseudotachylyte is uncommon in the most southerly outcrops and appears to be totally absent in the north. This corresponds both to an increase in the amount of available water and to a decrease in temperature and pressure going gradually up section in the direction of thrusting within the footwall [62,93]. In effect, the amount of pseudotachylyte decreases going upward in the crustal section toward the depth of the typical seismogenic zone at around 15–20 km [108].

### (c) Evidence of transient high stress in the shear zones

The occurrence of several overprinting cycles of ductile deformation and pseudotachylyte-producing, seismic brittle fracturing is reported across both the Woodroffe Thrust [54,61,62,93] (figure 3) and the DSZ [53,63,107]. Integrated field, microstructural and petrological investigations [53,107] have established that transient high stress repeatedly resulted in the formation of fractures and pseudotachylytes, providing fine-grained, weak, planar precursors that localized subsequent ductile shear zones (figures 4 and 5) under the same dry, high-grade conditions (approx. 600–650°C, 1.0–1.2 GPa, [63]). Fracturing under lower crustal dry conditions implies high deviatoric stresses, in the order of 1 GPa [107,109]. However, the grain size of quartz in mylonites from the DSZ is generally quite coarse (50–100 µm) but markedly variable, ranging from 1 to 200 µm. This has been interpreted to reflect correspondingly large variations in deviatoric stress, from 10 to 500 MPa or more [53]. Transiently, stresses could even have reached GPa levels, which may be necessary to explain extensional and shear fracturing of garnet [107] (figure 5*b*), intermittent seismic fracture of dry strong rocks, and pseudotachylyte development under lower crustal, sub-eclogitic facies conditions.

### (d) Generation of earthquakes in the Musgrave Range

The Woodroffe Thrust provides an almost unique record of a well-exposed section of middle to lower continental crust that can be studied over a distance of approximately 60 km in the direction of thrusting, showing the effects of a continuous gradient in temperature and fluid availability toward initially shallower levels. This exceptional record provides tight constraints on the seismic structure of the continental crust and on the mechanisms of generation of lower crustal earthquakes. The disappearance of pseudotachylyte toward the north in the footwall of the Woodroffe Thrust can be used as an argument against the origin of pseudotachylyte at the middle to lower crustal levels by downward propagation of aftershock swarms following major earthquakes in the seismogenically active upper crust [54]. Seismic attenuation during such downward migration should result in a decreasing abundance of pseudotachylyte from structurally higher to lower levels, that is from north to south in our profile, but in fact, the opposite is true and pseudotachylyte abundance is significantly greater in the dry (southern) than in the wet (northern) mylonites of the footwall. The footwall Mulga Park Subdomain was, as a result of the thrust geometry, originally at a higher crustal level than the Fregon Subdomain in the hanging wall. However, the Mulga Park Subdomain largely flowed during Petermann deformation, developing mylonitic shear zones and broad folds but very little pseudotachylyte. By contrast, the Fregon Subdomain both fractured, with widespread development of multiple generations of pseudotachylyte, and discontinuously flowed, with shear zones bounding large intervening low-strain domains. Across the Woodroffe Thrust, we, therefore, have an inverted section [93]: the Fregon subdomain must represent a seismogenic zone as it contains abundant pseudotachylyte but it was tectono-stratigraphically below the ductile zone represented by the Mulga Park Subdomain. Consequently, these pseudotachylytes cannot be produced by the downward propagation of earthquake ruptures. This observation suggests that a more local source was required for the widespread and abundant development of pseudotachylyte in the dry lower crustal Fregon Subdomain [53]. Transient stress concentrations, developed as the result of jostling between strong, low-strain blocks bounded by arrays of weaker shear zones (figure 4), were proposed to be the local trigger for seismic fracture and pseudotachylyte formation [53]. This argument is supported by rock-analogue and numerical models [110,111] showing that a shear zone network can only continue to accumulate bulk strain if the low-strain domains, bounded by the shear zones, also deform. For a power-law viscous rheology related to crystal-plastic dislocation creep, which is considered typical of the ductile middle to lower crust, a decrease in strain rate results in an increase in the effective viscosity. This can lead to a feedback effect where such blocks become progressively stronger, with slower strain rate increasing the effective viscosity, which further decreases the strain rate, and so on. This is similar to the tendency for isolated power-law inclusions in a weaker matrix to become progressively stronger until they behave as effectively rigid inclusions [112–115]. Such effectively rigid blocks that are geometrically required to deform must build up stress, amplified at points of interaction, until they eventually fail by fracturing. For confining pressures of *ca* 1.2 GPa during the generation of pseudotachylytes in the Musgrave Province [63], the differential stress for failure in a dry rock will be of the same order. Shear fracture under these conditions, potentially releasing stored elastic energy from relatively large, low strain and effectively elastic rigid blocks with a long Maxwell relaxation time because of the high effective viscosity, could trigger significant earthquakes locally sourced in the dry lower crust. Such repetitive events would then be the direct cause of the observed, widely distributed pseudotachylytes [63]. It should be noted that a corollary of this model is that the bulk, long-term strength of the lower crust is determined less by the network of weak shear zones than by the intervening strong, lower strain domains.

This basic model was also proposed for the wet middle crustal shear zone network in the Neves area of the Eastern Alps, Italy [116]. In this area, the shear zones themselves are very weak [113] but most of the volume of the meta-granodioritic rock forms a low-strain domain, which, from field observations, initially fractures before subsequently localizing viscous shear on those fractures. Under high pore-fluid pressure and lower confining pressure, the stress release associated with such fractures would be much lower than in the dry lower crust and, indeed, pseudotachylyte in the Neves area is rare, in marked contrast to its almost ubiquitous occurrence in the Fregon Subdomain of the Musgrave Ranges.

A characteristic of this model is that fracture precedes viscous flow in progressively cannibalizing the low-strain domains. A further characteristic is that viscous shear zones, developed on fracture precursors, ignore scattered weaker inclusions, which would not be the case for purely viscous deformation [117]. This effect is also observed in the Musgrave Ranges [53,83]. Localized shear zones only develop on elongate tabular layers of different rheology, such as finer-grained dolerite dykes and fractures, often marked by pseudotachylyte, both of which are very long relative to their width (figure 4), and ignore inclusions such as enclaves in granitoids or quartz-rich pods inherited from before the Petermann Orogeny.

## 3. The Lofoten-Vesterålen area (northern Norway)

The Lofoten-Vesterålen islands in northern Norway expose a lower crustal section consisting of Archaean to Palaeoproterozoic ortho- and paragneisses, intruded at 1.9–1.7 Ga by a suite of anhydrous Anorthosite–Mangerite–Charnockite–Granite (AMCG) plutons [118]. Pluton emplacement occurred under granulite facies conditions estimated at 750–800°C and 0.4–1.2 GPa [119]. The primary igneous texture and the anhydrous granulite facies mineral assemblages are generally well preserved in the AMCG suite.

**Figure 6.**
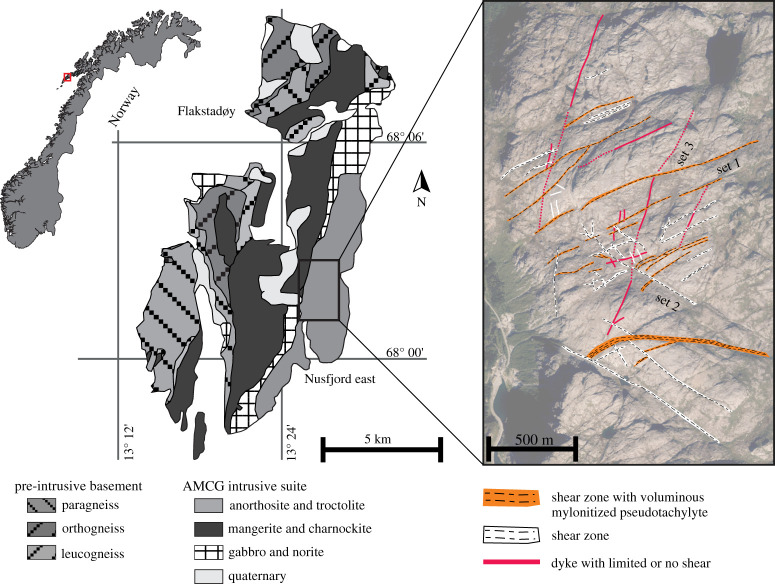
Geological map of Flakstadøy, Lofoten (after Steltenpohl *et al*. [72]), and aerial photo of the Nusfjord East anorthosite ridge illustrating the three major shear zone orientations. (Online version in colour.)

Lofoten-Vesterålen represent a tectonic window of basement rocks of the Baltica plate beneath the Caledonian nappe pile, which largely escaped the Caledonian tectono-metamorphic overprint due to the limited availability of fluids necessary to facilitate viscous deformation of the anhydrous, strong granulites [120]. Caledonian fabrics and structures are limited to localized ductile shear zones that developed under eclogite- and upper amphibolite to granulite facies conditions [51,72,121,122]. A number of studies from different localities in Lofoten-Vesterålen demonstrate that many of these high temperature shear zones exploit pseudotachylyte-bearing faults (*Eidsfjord and Fiskefjord*: [18,51,70,71,123]; *Nusfjord and Skagen*: [14,52,55,72,124]). In all these cases, pseudotachylytes and their mylonitized equivalents occur within anhydrous rocks such as anorthosites, monzonites and gabbronorites. In the following, we describe lower crustal pseudotachylytes and mylonites from the Nusfjord locality (figure 6) in the western part of the Lofoten-Vesterålen islands.

### (a) Pseudotachylytes in the Nusfjord East shear zone network

**Figure 7.**
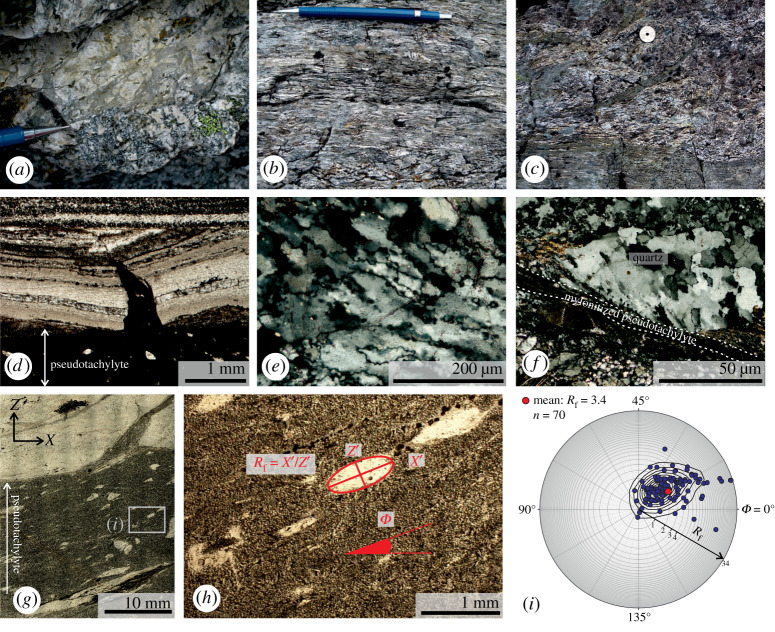
Nusfjord East shear zones and their microstructural evidence for transient high creep rates in the shear zone: (*a*) Weakly sheared pseudotachylyte breccia. The viscous overprint is identified from elongation and alignment of small clasts. (GPS location: WGS84 zone 33 W, 0431637 E, 7550356 N); (*b*,*c*) strongly sheared mylonitized pseudotachylyte with veins transposed toward parallelism with shear zone margin. Former clasts of the host anorthosite form elongate ribbons (white in colour). Outside the mylonitized pseudotachylyte in (*c*), the anorthosite shows little viscous deformation and injection veins of pseudotachylyte are only partially transposed. (GPS location: WGS84 zone 33 W, 0432042 E, 7549520 N); (*d*) pristine pseudotachylyte with injection vein cutting mylonitized pseudotachylyte (optical plane-polarized micrograph); (*e*) quartz aggregate microstructure showing bulging and subgrain rotation recrystallization (optical cross-polarized micrograph); (*f*) quartz aggregate displaying fine-grained recrystallized zone at margin of sheared pseudotachylyte (optical cross-polarized micrograph); (*g*) viscously sheared pseudotachylyte vein from same sample as (*e*) showing aligned, elongate clasts (optical plane-polarized micrograph). Rectangle indicates location of (*h*); (*h*) detail of elongate clasts showing determination of axial ratio (*R*_f_) and inclination (*Φ*) (optical plane-polarized image). Clasts are composed of recrystallized plagioclase or quartz aggregates plus minor pyroxenes; (*i*) results of *R*_f_–*Φ* analysis for clasts from vein shown in (*g*) plotted on Elliot polar plot (an equivalent *R*_f_–*Φ* graph is provided as electronic supplementary material figure S1). (Online version in colour.)

The Nusfjord East shear zone network is exposed within an E-W striking ‘high-strain zone’ of approximately 2 km^2^ on the ridge northeast of the village of Nusfjord (figure 6). The network consists of three main intersecting sets of ductile shear zones ranging in width from 1 cm to 1 m and dissecting granulitic anorthosites, gabbros, norites and charnockites. *Set-1* shear zones dip steeply mostly toward the S or SE and have normal-oblique kinematics; *set-2* shear zones are subvertical, strike NW-SE, and have a sinistral strike slip kinematics; *set-3* shear zones are subvertical, strike NNE-SSW, and have oblique normal kinematics. Mutual crosscutting relationships indicate that the three sets were active at the same time. All three sets contain mylonitized pseudotachylytes (*type-1 pseudotachylytes*, figure 7*a–c*), but these are, however, most extensively developed in the *set-1* shear zone orientation [52,55,124]. The three sets of shear zones separate relatively undeformed blocks of coarse-grained (grain size greater than or equal to 0.5 cm) anorthosite that contain pristine pseudotachylyte fault veins (*type-2 pseudotachylytes*). *Type-2 pseudotachylytes* link adjacent or intersecting ductile shear zones, and are interpreted as fossil seismogenic faults representing earthquake nucleation as a transient consequence of ongoing, localized aseismic creep along the shear zones [55]. *Type-2 pseudotachylytes* will be discussed further in §3c.

Pressure–temperature conditions of mylonitization of *type-1 pseudotachylytes* and of generation of *type-2 pseudotachylytes* were estimated at 650–750°C, 0.7–0.8 GPa [52] (table 1, figure 1). ^40^Ar-^39^Ar dating of localized upper amphibolite facies shear zones in the Nusfjord area with similar orientation to the three sets of shear zones in the Nusfjord East network yielded an age range of 433–413 Ma [121,122,125]. This suggests that the shear zones formed during the collisional (Scandian) stage of the Caledonian Orogeny, and that the brittle–viscous deformation cycles represent the rheological response of the Baltica basement underthrusting Laurentia. Further structural, petrological and geochronological work is required to confirm the tectonic setting of the Nusfjord East shear zone network.

The mylonitized *type-1 pseudotachylytes* consist of a fine-grained (average grain size: 5–30 µm) mixture of plagioclase (An_46-58_), amphibole (pargasite) and clinopyroxene (diopside), with minor amounts of garnet, biotite, quartz and K-feldspar. The transition from pseudotachylytes to localized (ultra)mylonitic shear zones was attributed to the activation of grain size sensitive creep in *type-1 pseudotachylytes* and their damage zone, which was facilitated by an increase in H_2_O content from the anorthosite host rock to the mylonitized pseudotachylytes (0.04 versus greater than or equal to 0.25 wt%, respectively, [52]). The aqueous fluid did not penetrate the interior of nominally anhydrous minerals (plagioclase and clinopyroxene) to potentially promote hydrolytic weakening [126]. Rather, the infiltrated H_2_O assisted the syn-kinematic nucleation of new phases (e.g. amphibole), which inhibited grain growth due to pinning with second phase particles and caused strain localization in the polymineralic, fine-grained mylonitized pseudotachylytes [52]. Extrapolation of laboratory-derived flow laws at the inferred deformation conditions of Nusfjord indicates a viscosity reduction of approximately 7 orders of magnitude during the formation of mylonitized pseudotachylytes deforming by diffusion creep compared to the dry anorthosite host [45,49,52,127]. While grain size sensitive creep in mylonitized pseudotachylytes may represent long-term aseismic creep during interseismic stages, the microstructure in these shear zones locally indicates transient oscillations in strain rate and stress that are likely to result from the earthquake cycle deformation.

### (b) Evidence of transient high creep rates in the shear zones

The spatial association of mylonites and pseudotachylytes inherently suggests cycling of stresses and strain rates over some period of time. This is especially true where repeated generations of pseudotachylyte alternate with viscous creep (figure 7*d*). If, in addition, the seismicity represented by the pseudotachylytes and the aseismic creep represented by the mylonitization can be shown to be broadly coeval, then strain rate variations might have occurred on the timescale of earthquake cycle deformation.

Some *type-1* mylonitized pseudotachylytes in the Nusfjord east shear zones have been shown to preserve a highly localized microstructural record of transient high-strain rate creep [124], in addition to the wider record of longer-term grain-size sensitive creep in the same mylonitized pseudotachylyte shear zones [52]. Within some mylonitized pseudotachylyte veins (figure 7*e*) or in the host rock immediately adjacent to them (figure 7*f*), monomineralic ribbons of quartz show a mixed population of larger, strained grains, alongside more equant finer grains. At sites at the vein margin, the ratio of finer grains to large grains increases with proximity to the pseudotachylyte (figure 7*f*), whereas in quartz ribbons within a vein the finer grains mantle the larger grains (figure 7*e*). EBSD analysis indicates that the finer grains have low values of grain orientation spread (GOS, a proxy for internal strain in each grain) and have the same *c*-axis crystallographic preferred orientation (CPO) as the larger grains. These finer grains are interpreted to have formed via bulging and subgrain rotation recrystallization. The recrystallization occurred at the same high *P*–*T* conditions of mylonitization of *type-1 pseudotachylytes*, as indicated by the coexistence of fine-grained recrystallized quartz with recrystallized clinopyroxene [124]. Fine-grained recrystallized quartz is only found immediately adjacent to, or sitting within, a mylonitized pseudotachylyte vein. At more distal sites relative to the pseudotachylyte veins, quartz recrystallization occurs at a much reduced extent (if at all) and generates a larger recrystallized grain size, implying that the finer-grained microstructure represents deformation conditions localized along the pseudotachylyte veins.

Quartz piezometry [128] applied to the fine-grained low-GOS recrystallized quartz population resulted in a mean flow stress of 98 ± 18 MPa [124]. At temperatures of approximately 700°C, this is equivalent to strain rates of 10^−9^ s^−1^ using a two-phase area-weighted mixed flow law combining quartz dislocation creep [129,130] and anorthite–diopside diffusion creep [131]. These two flow laws were combined to model the rheology of the mylonitized pseudotachylytes based on the microstructural observations and EBSD analysis of recrystallized monophase quartz ribbons deforming by dislocation creep and embedded in a fine-grained, plagioclase + clinopyroxene-rich aggregate deforming by diffusion creep [52,124].

The effective viscosity of the quartz-bearing mylonitized pseudotachylyte deforming at 10^−9^ s^−1^ is approximately 10^16^ Pa s at 700°C [124]. This strain rate is high relative to typical geological strain rates [132]. The high-strain rate creep is localized to the mylonitized pseudotachylyte vein and its margins, and is not observed in the margin of pristine, non-mylonitized pseudotachylytes. Thus, it is interpreted to have occurred after the coseismic slip that initially generated the pseudotachylyte, and to be temporally transient within the long-term viscous deformation of the Nusfjord East shear zones. The transient high-strain rate is not preserved more widely across the mylonitized pseudotachylyte veins due to overprinting by lower stress and strain rate creep. Evidence for a lower stress overprint of the high stress recrystallized quartz consists of foam textures with larger, low-GOS quartz grains with common 120° triple junctions of grain boundaries [124]. Similar quartz foam textures have been interpreted to form following rapid decreases in differential stress [133].

The transient high-strain rate creep recorded by the quartz microstructure could represent post-seismic deformation, because this fast rate of deformation is both within the range of post-seismic strain rates and not easily attributable to any other geological processes [132]. The proposed viscosity of this high-strain rate deformation is similar to geodetic observations of post-seismic relaxation in the lower crust [124]. The timescale of post-seismic relaxation of elevated stresses and strain rates [20,134,135] is geologically short. Pseudotachylyte cooling and crystallization [136–138], followed by solid-state recrystallization and grain growth of the foam texture quartz [133,139,140], could feasibly all occur at 700°C within a viable post-seismic period of approximately 100 years [124].

A relevant question is whether enough strain could be accumulated during post-seismic relaxation in order to drive the microstructural evolution of quartz. The strain associated with the transient microstructures is not necessarily easy to constrain against the background of long-term lower strain rate viscous creep. However, the noted tendency of quartz microstructure to respond rapidly to reductions in stress [133] might allow some estimate of post-seismic strain to be constrained. For example, figure 7*g* illustrates a mylonitized pseudotachylyte vein with aligned clasts and ribbons composed of plagioclase and quartz aggregates; an example of the latter was analysed from this sample for quartz piezometry [124]. No foam texture style microstructures, indicative of reduced stresses, were observed in this sample, and the recrystallized quartz grain size was consistent with others used to derive the high stresses and strain rates in the Nusfjord mylonitized pseudotachylytes [124]. Assuming that the clasts in the pseudotachylyte prior to viscous solid-state deformation were randomly oriented, an *R*_f_–*Φ* analysis can be carried out by assessing the axial ratio of the major and minor axes lengths for each clast, plus the orientation of the major axis (figure 7*h*). The results for a population of 70 clasts from across the vein shown in figure 7*g* are shown on an Elliot polar plot (figure 7*i*). The mean value suggests a strain of approximately 3.4, broadly consistent with converting the mean inclination of the clasts (defining the internal shear zone foliation) from the shear zone boundary—which, at 19.5°, is equivalent to a strain of 2.5. Assuming an approximate shear strain value of 3 and a strain rate of 10^−9^ s^−1^, the required time for strain accumulation would be approximately 95 years, on the same magnitude as post-seismic timescales. The equivalent displacement, given an average width for this mylonitized pseudotachylyte vein of 2 cm (see fig. 2*a* of [124]) and assuming homogeneous strain across the width, is 9 cm. The Nusfjord East *type-1 pseudotachylytes* are, therefore, one of the first cases that can promote pseudotachylytes as a viable rheology capable of supporting post-seismic relaxation within the lower crust, and suggest that the post-seismic component of displacement could be relatively significant.

### (c) Generation of earthquakes in Nusfjord by local stress amplifications

**Figure 8.**
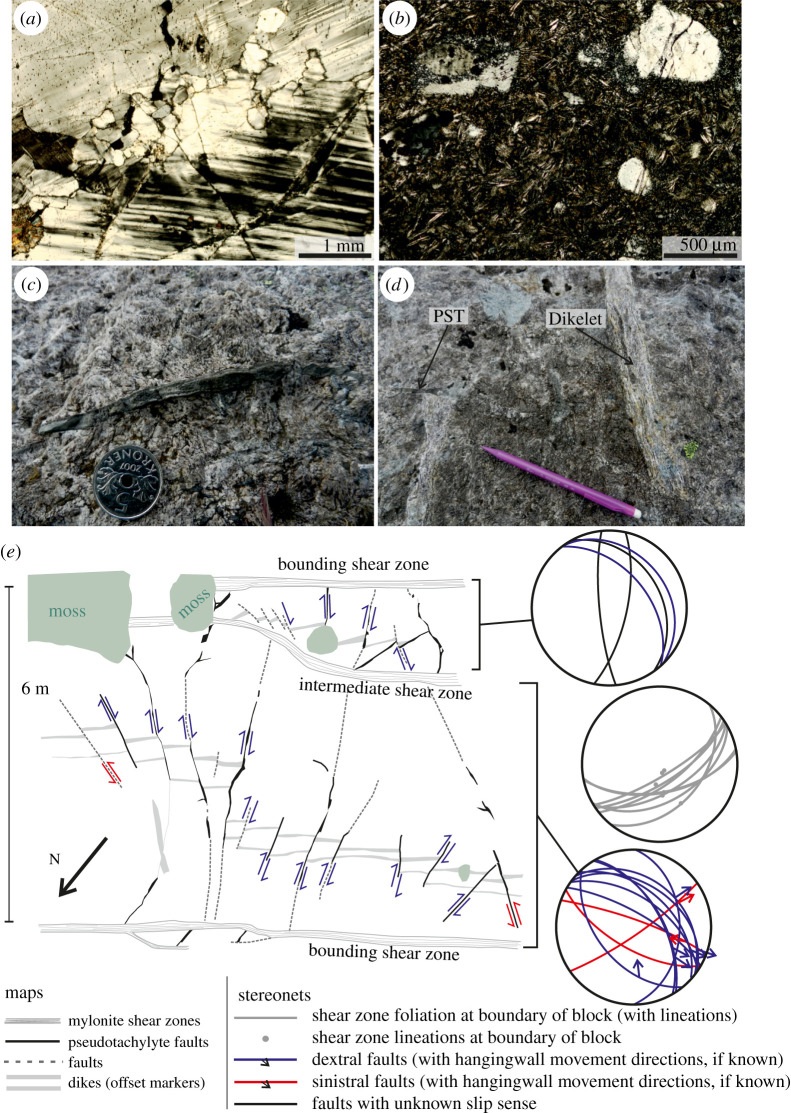
Generation of earthquakes due to stress amplification: (*a*) microfractured anorthosite from interior of shear zone bounded block (optical cross-polarized image); (*b*) elongate, skeletal and/or radiating plagioclase microlites and embayed and rounded survivor clasts are some of the evidence used to identify non-mylonitized *type-2 pseudotachylytes*; (*c*) *type-2 pseudotachylyte* vein with chilled margins and flow banding; (*d*) *type-2 pseudotachylyte* vein (PST) with apparent dextral fault offset of anorthosite dikelets; (*e*) sketch map of *type-2 pseudotachylyte* network developed in an anorthosite block with bounding and intermediate shear zones. Orientations of the pseudotachylyte faults and their bounding shear zones are shown. (Online version in colour.)

The Nusfjord East shear zone network provides outstanding field examples, supplementing recent work in the Musgrave Ranges [53], on which to examine the controls of earthquake nucleation in dry lower crust. In the anorthosite hosted shear zones of Nusfjord East, small length-scale pseudotachylyte fault veins are intimately connected with the geometry of the localized shear zone network. The varied orientations of shear zones in the Nusfjord East network creates a blocky and irregular geometry to the low-strain anorthosite sections lying between the shear zones [55]. For the most part, these anorthosite blocks remain coarse-grained, dry and resistant to viscous shear. Microcracking is, however, prevalent in some samples (figure 8*a*).

Between closely spaced adjacent shear zones, pseudotachylyte veins are observed cutting across the anorthosite blocks. These pseudotachylytes (*type-2 pseudotachylytes*) are typically distinguished from the sheared *type-1 pseudotachylytes* by their common preservation of primary pseudotachylyte features, including quench crystallization morphologies and randomly orientated clasts (figure 8*b*), by chilled margins and flow structures (figure 8*c*) and by preserved planar fault geometries such as stepovers. These pseudotachylyte veins are identified as fault (generation-plane) veins via the offset of small dykelets across them (figure 8*d*).

*Type-2 pseudotachylyte* faults are found in networks where cross cutting faults display different senses of displacement. An example of such a network is shown in figure 8*e*; others are displayed in [55]. In figure 8*e*, the anorthosite block is bounded by two SE-dipping *set-1* shear zones, with an intermediate shear zone cutting through the block on the southern side. Faults with pseudotachylyte may cut across the entire block, but do not continue across the bounding or intermediate shear zones. Neither are the pseudotachylyte faults cut and displaced by the shear zones—at the locality shown in figure 8*e*, the pseudotachylyte faults to the south of the intermediate shear zone appear to be part of a separate network from those developed in the northern section, with no obvious continuity between the faults networks on either side. Many of the faults across the locality are apparently dextral, and these typically dip moderately to the northeast (figure 8*e*). Where the slip direction can be determined using two or more offset dykelets, these apparent dextral faults typically show dextral oblique normal slip. Sinistral faults are steeper, dipping south-eastward and south-westward, and show slightly reverse-oblique sinistral slip (figure 8*e*). The maximum fault-length measured in this locality is 4.3 m. This network of cross cutting pseudotachylyte faults restricted to an anorthosite block between shear zones is repeated in several other localities across the Nusfjord east shear zone network, with the width of the block they cut ranging from approximately 1 m up to 15 m [55]. The orientation of the *type-2 pseudotachylyte* faults varies with the geometry of the bounding shear zones, but there is some consistency between spatially associated blocks with similar orientations (compare fig. 8e to fig. 3a of [55]).

Displacements and lengths of *type-2 pseudotachylyte* faults are well constrained in several examples of these shear zone bounded blocks. The *type-2* faults are typically segmented, displaying pseudotachylyte-filled stepovers that provide a record of single-slip seismic displacement measured along the fault-parallel sides of the pull-apart [141]. This method was chosen in order to avoid any risk of including a viscous component that potentially would increase displacements calculated from offset passive markers such as dykelets. The main uncertainty of this method arises from it being potentially a measure of apparent displacement only. In most of the cases, the actual slip vector could be calculated where two or more passive markers are cut by the same *type-2* fault, using the separation, orientation and offset of those markers [124]. In a few cases, the length of pull-apart was measured along *type-2* faults that lack any passive marker, thus making such measurements of coseismic slip less well constrained. Displacements range from 0.01 to 0.26 m [55]. By assuming a realistic range of fault geometries (i.e. circular to elliptical), the earthquake moment for the events that generated these *type-2 pseudotachylytes* could be calculated and converted to a moment magnitude, giving a range for these faults of *M*_w_ 0.2–2.6 using rupture area aspect ratios of 1–10 [55]. Static stress drops could also be calculated using the ratio between the fault size and the displacement [142]; these range between 0.06 and 4.2 GPa for the *type-2 pseudotachylytes*, considering the same range of fault aspect ratios [55].

These observations were interpreted to show small-scale earthquake ruptures that nucleated within the lower crust [55]. The ruptures appear to relate to the failure of the low-strain anorthosite blocks in relation to coeval viscous creep along the bounding shear zones. This places the temperature and pressure conditions of seismicity at the same deformation conditions as the mylonitized *type-1 pseudotachylytes* in the bounding shear zones at 650–750°C and 0.7–0.8 GPa [52]. This is supported by the stability of the granulite facies mineral assemblage (plagioclase + clinopyroxene + hornblende + orthopyroxene + garnet + biotite ± quartz ± K-feldspar) and by the absence of greenschist facies alteration (e.g. epidote, chlorite, albite) in the damage zone of *type-2 pseudotachylytes* and in the veins themselves [55].

The high static stress drops estimated for the *type-2 pseudotachylytes* are much higher than those typically estimated for earthquakes in the seismogenic zone (0.1–100 MPa, [143]), including earthquakes of similar small magnitudes which are sometimes considered to have ‘high’ stress drops (closer to 100 MPa, e.g. [144,145]). They also extend to an order of magnitude higher than stress drop calculations for lower crustal and upper mantle earthquakes at larger magnitudes [146,147]. Thus, the discrepancy between our calculations and the seismological estimates must be addressed. In high-speed rotary friction experiments that simulate earthquakes slip velocities (1 ms^−1^), frictional wakening due to melting is dramatic [148]. Triaxial experiments, where pseudotachylytes were formed during spontaneous stick-slip, showed stress drops of 100% of the peak stress at confining pressure above 300 MPa [149]. However, the dramatic increase in stress drop that should arise from thermal weakening is not observed in seismological data [150]. Thus, it appears that the stress drops estimated from pseudotachylyte-bearing faults are unusual for earthquakes, and that pseudotachylytes are not representative of average fault shear strength during the earthquake that generated them [151]. Field studies of pseudotachylyte-bearing faults in Kings Canyon national park (California) [152] concluded that the stress drop over the patches of the faults that melted was complete and that this must have been balanced by low-stress drops in the non-melted portions of the faults. The discrepancy between the estimate of stress drops from pseudotachylytes and those determined by seismological means may thus arise from the patchy distribution of pseudotachylytes along large ruptures. In the case of the *type-2 pseudotachylytes* at Nusfjord, the dimensions of the surface rupture was limited to the size of the block and most of the surface rupture was lined with pseudotachylytes. This might have allowed for an almost complete stress drop, of the same order of magnitude of the failure shear strength of the intact anorthosite, which at the depth inferred at Nusfjord must be greater than 1 GPa [55]. Higher stress drops (e.g. 4.2 GPa) might indeed be unrealistically high, and presumably represent cases where the length of the pull-apart has overestimated the single-slip seismic displacement considerably, probably due to sectioning reasons.

Stress drops on the order of GPa, consistent with the expected failure strength of intact anorthosite at lower crustal depths, imply that stresses in the anorthosite block needed to be locally amplified to such levels in order to facilitate failure. Local stress amplifications in the anorthosite blocks could be generated as a signature of strain incompatibility between the viscously deforming shear zones and the stronger anorthosite blocks [124]—such stress concentrations are modelled for systems with two contrasting viscosity materials, including shear zones [111] and melanges [153]. However, the magnitude and record of this proposed stress amplification still requires further constraints, which could feasibly be recovered using additional techniques such as numerical modelling, elastic geobarometry and high-angular resolution electron backscatter diffraction (HR-EBSD).

## 4. Final remarks and future directions

The Musgrave Ranges (Central Australia) and Nusfjord Area (Lofoten, Northern Norway) are comparable to the extent that dry protolith has been locally overprinted in a much later (hundreds of millions of years later) event by shear zones developed in the middle to lower crust (25–40 km depth). In both cases, the shear zones predominantly exploited pseudotachylyte-bearing faults that demonstrably developed under the same high-grade metamorphic conditions. While this observation reinforces the concept that products of lower crustal earthquakes represent agents of rheological weakening in otherwise dry and strong lower crust, an important difference between the two localities emerges. In Nusfjord, there is a clear spatial association between the deformation of pseudotachylytes and fluid infiltration [52], whereas both formation and deformation of pseudotachylytes in the granulitic hanging wall of the Woodroffe Thrust in the Musgrave Province occurred under dry conditions [61,63,93]. The studies in the Musgrave Ranges demonstrate that the transition from brittle seismic slip to viscous aseismic creep in the dry lower crust does not necessarily require fluid infiltration and hydration reactions, and suggests that the extreme grain size reduction and phase mixing associated with the generation of pseudotachylyte is capable of driving rheological weakening on its own, presumably through the activation of grain size sensitive creep. The earthquake-induced introduction of H_2_O into the dry and strong lower crust will amplify the weakening effect resulting from the dramatic grain size reduction and phase mixing [12,14].

In both localities, the microstructure of quartz in the mylonites is consistent with high stress and strain rate transients during non-steady-state viscous creep [53,107,124] (figure 7*e*,*f*). These high stress and strain rate creep transients could have occurred during the same seismic cycle that initially generated the pseudotachylyte, or could have occurred at a later stage associated with some other loading or unloading event. The timescale necessary to drive the microstructural evolution of quartz from fine recrystallized grains indicative of transient high-strain rate to coarser aggregates indicative of lower differential stress is compatible with the seismic cycle [124]. In either case, it is apparent that the production of pseudotachylyte in the lower crust has an important rheological effect on the ability of dry, strong lower crust not only to initiate localized viscous creep processes [21,52], but also to support rapid changes in stress and strain rate. This is a critical observation in the evaluation of the role the lower crust might play in the post- and interseismic phases of the seismic cycle [135,154,155].

The spatial distribution of pseudotachylytes both in the Woodroffe Thrust and in the Nusfjord area is consistent with a local (deep) source for the lower crustal earthquakes. This deep source may be triggered by transient, localized stress concentrations developed due to jostling and interaction of strong, low-strain domains within a network of actively deforming ductile shear zones [53,55]. Repeated cycles of pseudotachylyte generation over geological time may weaken considerable portions of the otherwise dry and strong lower crust, so that both the long-term and the short-term rheological weakening of the lower crust are controlled by deformation during the earthquake cycle. However, as long as low-strain domains of strong, rigid material are volumetrically dominant, it is their deformation by seismic fracturing as a precursor to new shear zone development that will determine the bulk rheology.

While the proposed model of a local origin for lower crustal seismicity by localized stress amplification is supported by the spatial distribution of pseudotachylytes, it must be demonstrated that stress amplification of the required magnitude can be achieved. Future work should make use of micro-analytical techniques capable of estimating residual stresses in geological material, such as HR-EBSD to determine the state of stress of deep seismogenic faults. Numerical models benchmarked to the geometry, kinematics and rheology of the shear zone networks in Nusfjord and in the Musgrave Ranges will also be valuable ways to estimate the magnitude of heterogeneous and localized stress amplification in a shear zone network [156].

Finally, while the earthquake-induced weakening of the lower crust through the pseudotachylyte-(ultra)mylonite transformation is well documented [45,49,52], it remains to be established why ultramylonites (generally considered to be weak) are locally overprinted by still younger generations of lower crustal pseudotachylytes, as frequently observed both in Nusfjord [52] and in the Musgrave Ranges [61–63]. Are the ultramylonites undergoing progressive hardening, or might thermal runaway be responsible for the generation of new pseudotachylytes? Or do the new generations of pseudotachylyte represent seismic fractures that initiated at stress concentrations within the adjacent rigid blocks, which then locally followed pre-existing weakened shear zones? Answering these questions will shed new light on the seismic behaviour of the lower crust, and will continue to require detailed field observations and high-resolution microstructural analysis of associations of coeval pseudotachylytes and mylonites, integrated with numerical modelling and experimental work.

## Supplementary Material

Strain analysis of mylonitised pseudotachylyte from Nusfjord

## Data accessibility

This review paper does not include new primary data, except the strain analysis of the mylonitized pseudotachylyte from Nusfjord reported in figure 7*i*. The strain analysis data have been uploaded into the electronic supplementary material.
