# Supplementary material for: The earthquake cycle in the dry lower continental crust: insights from two deeply exhumed terranes (Musgrave Ranges, Australia and Lofoten, Norway)
Source: Philos Trans A Math Phys Eng Sci. 2021 Feb 1;379(2193):20190416. doi: 10.1098/rsta.2019.0416 (PMC7898122; doi:10.1098/rsta.2019.0416)
Supplement: Strain analysis of mylonitised pseudotachylyte from Nusfjord [file rsta20190416supp1.doc]

# The earthquake cycle in the dry lower continental crust: insights from two deeply exhumed terranes (Musgrave Ranges, Australia and Lofoten, Norway)

| **Clast ID** | **R_f_: axial ratio** | **Φ: Inclination from horizontal (°)** | **Equivalent shear strain from inclination** |
| --- | --- | --- | --- |
| **1** | 4.662 | 21.96 | 2.1 |
| **2** | 6.205 | 16.8 | 3.0 |
| **3** | 4.559 | 9.96 | 5.5 |
| **4** | 13.954 | 10.6 | 5.2 |
| **5** | 3.679 | 28 | 1.3 |
| **6** | 2.649 | 24 | 1.8 |
| **7** | 3.785 | 19 | 2.6 |
| **8** | 1.566 | 10 | 5.5 |
| **9** | 2.413 | 9 | 6.2 |
| **10** | 3.075 | 15 | 3.5 |
| **11** | 3.747 | 16 | 3.2 |
| **12** | 1.151 | 9 | 6.2 |
| **13** | 6.789 | 23 | 1.9 |
| **14** | 3.429 | 28 | 1.3 |
| **15** | 2.975 | 19 | 2.6 |
| **16** | 2.165 | -3 | -19.0 |
| **17** | 2.342 | 24 | 1.8 |
| **18** | 2.125 | 34 | 0.8 |
| **19** | 4.980 | 22 | 2.1 |
| **20** | 1.957 | 51 | -0.4 |
| **21** | 7.172 | 16 | 3.2 |
| **22** | 2.491 | 21 | 2.2 |
| **23** | 2.257 | 21.5 | 2.1 |
| **24** | 3.817 | 26 | 1.6 |
| **25** | 3.675 | 7 | 8.0 |
| **26** | 3.123 | 23 | 1.9 |
| **27** | 2.714 | 41 | 0.3 |
| **28** | 2.595 | 24 | 1.8 |
| **29** | 2.715 | 23 | 1.9 |
| **30** | 4.034 | 24 | 1.8 |
| **31** | 10.441 | 12 | 4.5 |
| **32** | 3.432 | 24 | 1.8 |
| **33** | 2.076 | 28 | 1.3 |
| **34** | 2.568 | 21 | 2.2 |
| **35** | 2.158 | 15 | 3.5 |
| **36** | 3.375 | 28 | 1.3 |
| **37** | 2.204 | 12.5 | 4.3 |
| **38** | 5.292 | 7 | 8.0 |
| **39** | 3.634 | 21 | 2.2 |
| **40** | 2.815 | 17 | 3.0 |
| **41** | 1.975 | 30 | 1.2 |
| **42** | 2.250 | 33 | 0.9 |
| **43** | 1.717 | 20 | 2.4 |
| **44** | 4.189 | 24 | 1.8 |
| **45** | 1.776 | 17 | 3.0 |
| **46** | 2.173 | 29 | 1.2 |
| **47** | 2.250 | 23 | 1.9 |
| **48** | 5.333 | 28 | 1.3 |
| **49** | 1.347 | -48 | 0.2 |
| **50** | 3.022 | 22 | 2.1 |
| **51** | 3.407 | 19 | 2.6 |
| **52** | 5.895 | 16 | 3.2 |
| **53** | 11.596 | 13 | 4.1 |
| **54** | 3.830 | 29 | 1.2 |
| **55** | 2.224 | 32 | 1.0 |
| **56** | 3.836 | 28.7 | 1.3 |
| **57** | 3.852 | 19.31 | 2.5 |
| **58** | 1.133 | -34 | -0.8 |
| **59** | 5.180 | 19.42 | 2.5 |
| **60** | 3.750 | 18.1 | 2.7 |
| **61** | 9.814 | 16.5 | 3.1 |
| **62** | 3.397 | 28.5 | 1.3 |
| **63** | 3.685 | 31 | 1.1 |
| **64** | 2.154 | 31.5 | 1.0 |
| **65** | 5.150 | 33.1 | 0.9 |
| **66** | 3.533 | 16.2 | 3.2 |
| **67** | 4.681 | 22.2 | 2.0 |
| **68** | 1.824 | 25.4 | 1.6 |
| **69** | 2.464 | 10.5 | 5.2 |
| **70** | 3.339 | 14.9 | 3.5 |
| **MEAN** | **3.737** | **19.5** | **2.2** |
| **MAX** | **13.954** | **51** | **8.0** |

**Supplementary Table 1: M**easurements from clasts shown in Figs. 3h-i, used for R_f_ – Φ analysis (Fig. 3j and Supp. Fig. 1)


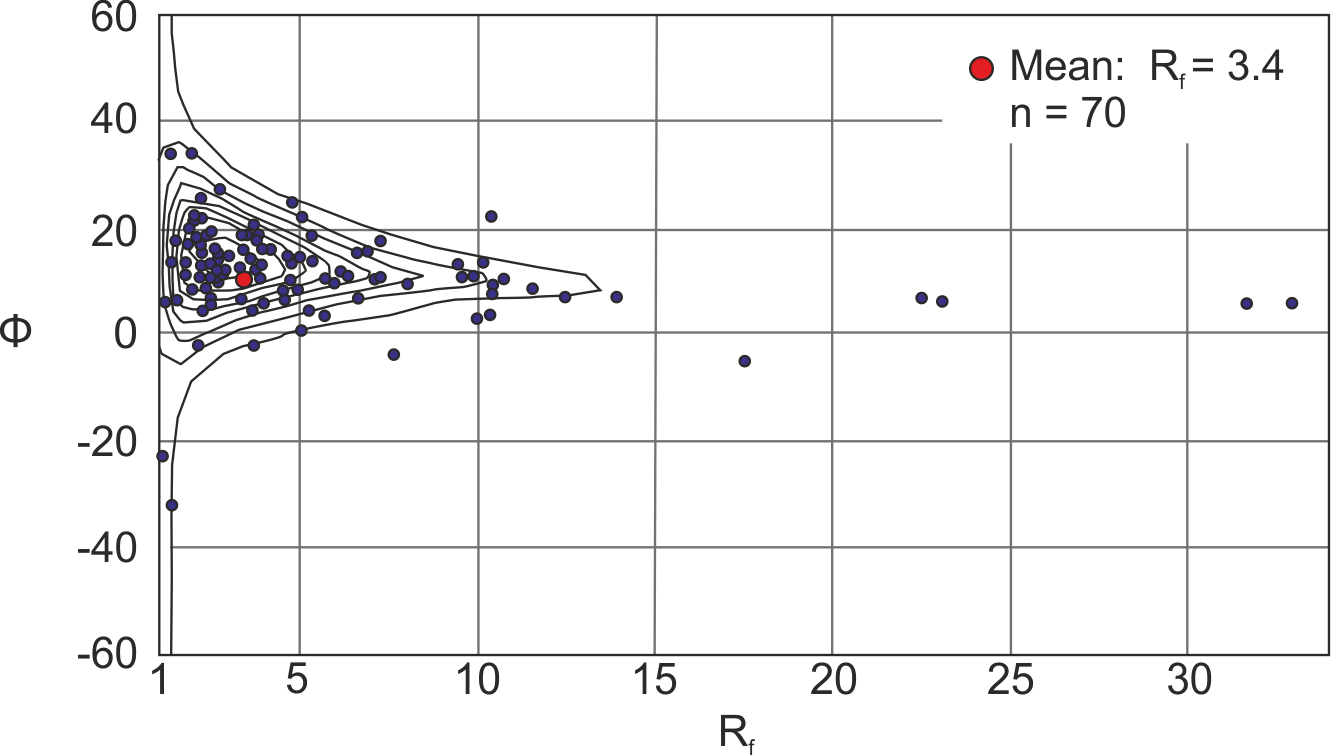


**Supplementary Figure 1:** R_f_ - Φ plot for clast measurements from Supplementary Table 1..
